# Identification of key biomarkers in steroid-induced osteonecrosis of the femoral head and their correlation with immune infiltration by bioinformatics analysis

**DOI:** 10.1186/s12891-022-04994-7

**Published:** 2022-01-18

**Authors:** Jun Zhao, Xingshi Zhang, Junjie Guan, Yu Su, Jizhao Jiang

**Affiliations:** 1 Zhuhai Hospital of Integrated Traditional Chinese and Western Medicine, No.208 Yuehua Road, Gongbei, Zhuhai, Guangdong China; 2 Harbin Fifth Hospital, Jiankang Road, Xiangfang District, Harbin, Heilongjiang China

**Keywords:** Steroid-induced osteonecrosis of the femoral head, Diagnostic, Immune cells, CIBERSORT, Bioinformatics analysis

## Abstract

**Objective:**

This study aimed to identify key diagnostic markers and immune infiltration of (SONFH) by bioinformatics analysis.

**Methods:**

Related SONFH datasets were downloaded from the Gene Expression Omnibus (GEO) database. First, we identified the differentially expressed genes (DEGs) and performed the functional enrichment analysis. Then weighted correlation network analysis (WGCNA) and the MCODE plug-in in Cytoscape were used to identify the diagnostic markers of SONFH. Finally, CIBERSORT was used to analyze the immune infiltration between SONFH and healthy controls, and the correlation between infiltrating immune cells and diagnostic markers was analyzed.

**Results:**

TYROBP, TLR2, P2RY13, TLR8, HCK, MNDA, and NCF2 may be key diagnostic markers of SONFH. Immune cell infiltration analysis revealed that Memory B cells and activated dendritic cells may be related to the SONFH process. Moreover, HCK was negatively correlated with CD8 T cells, and neutrophils were positively correlated with those key diagnostic markers.

**Conclusions:**

TYROBP, TLR2, P2RY13, TLR8, HCK, MNDA, and NCF2 may be used as diagnostic markers of SONFH, and immune-related mechanism of SONFH and the potential immunotherapy are worthy of further study.

**Supplementary Information:**

The online version contains supplementary material available at 10.1186/s12891-022-04994-7.

## Introduction

Osteonecrosis of the femoral head (ONFH) is a debilitating clinical disease characterized by the progressive necrosis of the bone marrow and bone cells, and most patients eventually develop progressive femoral head collapse and degenerative arthritis [1]. Although the etiology of ONFH remains unclear [2], many studies have confirmed the causal relationship between the steroid and the disease [3]. In Japan, 51% of patients with ONFH are due to systemic steroid administration, and the younger the age, the higher the proportion of SONFH and hip replacement [4], which brings a substantial economic burden to patients. Therefore, early detection is crucial for the successful treatment of SONFH. However, SONFH can be completely asymptomatic and only be detected by MRI (Magnetic Resonance Imaging) as early as possible [5]. Although MRI can diagnose early-stage SONFH, histological damage to the joint is earlier than the appearance of clinical symptoms [6]. Therefore, the exploration of biomarkers that are easy and early to diagnose is essential for treating SONFH patients.

The specific pathogenesis of SONFH remains unclear, but recent studies have revealed that immune cell infiltration associate with its occurrence and development. Studies demonstrated that the frequency of activated B cells in peripheral blood of SONFH was higher than healthy controls, and the degree of the femoral head collapse was positively correlated with the percentage of CD86 + CD19 + B cells [7]. Ma et al. [5] found that immunomodulatory cells, especially inhibitory T lymphocytes, which mainly regulate the bone mass balance of the femoral head by secreting a variety of cytokines such as osteoprotegerin and interleukin-4, are strictly related to the pathogenesis of nontraumatic ONFH. Moreover, Tian et al. [8] confirmed the disruption of immune response plays a role in the pathogenesis of SONFH. Therefore, from the view of the immune system, evaluating the differences in the composition of immune infiltrating cells in SONFH is of great value to elucidating its molecular mechanism and identifying molecular markers related to immune infiltration.

CIBERSORT (https://cibersort.stanford.edu/) algorithm is mainly used to calculate the proportion of immune cells in samples and has been used to analyze immune cell infiltration in many diseases, such as knee osteoarthritis [9], colorectal cancer [10], and idiopathic pulmonary fibrosis [11]. However, there is no study on immune cell infiltration related to SONFH. In our study, we downloaded the SONFH dataset from GEO database and identified the diagnostic markers by bioinformatics analysis. Then, the difference of immune infiltration was analyzed using CIBERSORT, and the relationship between immune cells and diagnostic markers was assessed to better clarify the molecular mechanism related to immunity during the development of SONFH.

## Materials and methods

### Data collection and preprocessing

GSE123568 (deposited by Taixian Li et al. [12]) and GSE26316 (stored by Peijian Tong et al. [13]), were downloaded from the GEO database (https://www.ncbi.nlm.nih.gov/geo/). GSE123568 included 40 serum samples, 30 SONFH and ten non-SONFH samples, and related clinical data, and the platform is Affymetrix Human Gene Expression Array. The validated dataset GSE26316 contains three femoral head from SONFH and three normal samples, and its platform is [Rat230_2] Affymetrix Rat Genome 230 2.0 Array.

According to the annotation information of the GPL15207 and GPL1355 platform, the data probes were transformed to gene symbols. Then, the associated package of R (version 4.0.2) was performed to preprocess these data, including background correction, add the missing values, and quantile normalization [14].

### Identification of DEGs, gene ontology (GO) enrichment analysis and gene set enrichment analysis (GSEA)

Differentially expressed mRNAs were identified by Limma package [15] in R with the cut-off criteria of *P*-value of < 0.05 after adjustment, and |log2FC| > 1. GO enrichment analysis on DEGs and GSEA on the gene expression matrix were performed using the “clusterProfiler” package [16], and “c2.cp.reactome.v7.1.entrez.gmt” was used as the reference gene set of GSEA [17]. A false discovery rate (FDR) < 0.25 and *p* < 0.05 were considered significant enrichment.

### WGCNA analysis to screen specific modules

The WGCNA R package was adopted to construct the co-expression network [18]. First, outlier samples were removed by sample clustering analysis. Second, the weighting coefficient β was determined according to the principle of the scale-free network. Third, the modules were detected using the dynamic tree cut algorithm with a minimum module size of 30. Fourth, the module membership (MM) and gene significance (GS) of the key module related to clinical attributes were calculated.

### Screening key diagnostic markers

The construction of the PPI (protein-protein interaction) network was based on the DEGs by STRING 11.0 (https://string-db.org) [19]. Besides, we used the MCODE (Molecular complex Detection) plug-in [20] in Cytoscape 3.7.1 to filter modules in the PPI networks with the MCODE score > 5 according to the default settings. For genes in the candidate module of WGCNA, a gene with MM > 0.9 and GS > 0.2 was defined as the hub gene. Finally, the key diagnostic markers obtained by the two algorithms were overlapped.

### Immune infiltration by CIBERSORT analysis

To obtain the immune cell matrix, we used the CIBERSORT algorithm to analyze the normalized gene expression matrix and filtered the samples with p<0.05. Then, the “corrplot” package (https://github.com/taiyun/corrplot) in R was used to calculate the correlation between immune cells and visualize it. Finally, the “vioplot” package in R was used to analyze the different levels of immune infiltration of each immune cell between the SONFH and non-SONFH groups.

### Correlation analysis between infiltrating immune cells and key diagnostic markers

The cor function in R language was used to calculate the Spearman correlation analysis between infiltrating immune cells and key diagnostic markers, and the result was visualized using the “corrplot” package.

### Receiver operating characteristic (ROC) analyses

Using GraphPad Prism 8.0 (GraphPad Software, Inc.), we carried out the ROC analyses to assess the diagnostic value of key diagnostic markers. The area under the curve (AUC) was calculated to evaluate the significance in dataset GSE26316, and the ROC curve was generated.

## Results

### Identification of DEGs

**Fig. 1:**
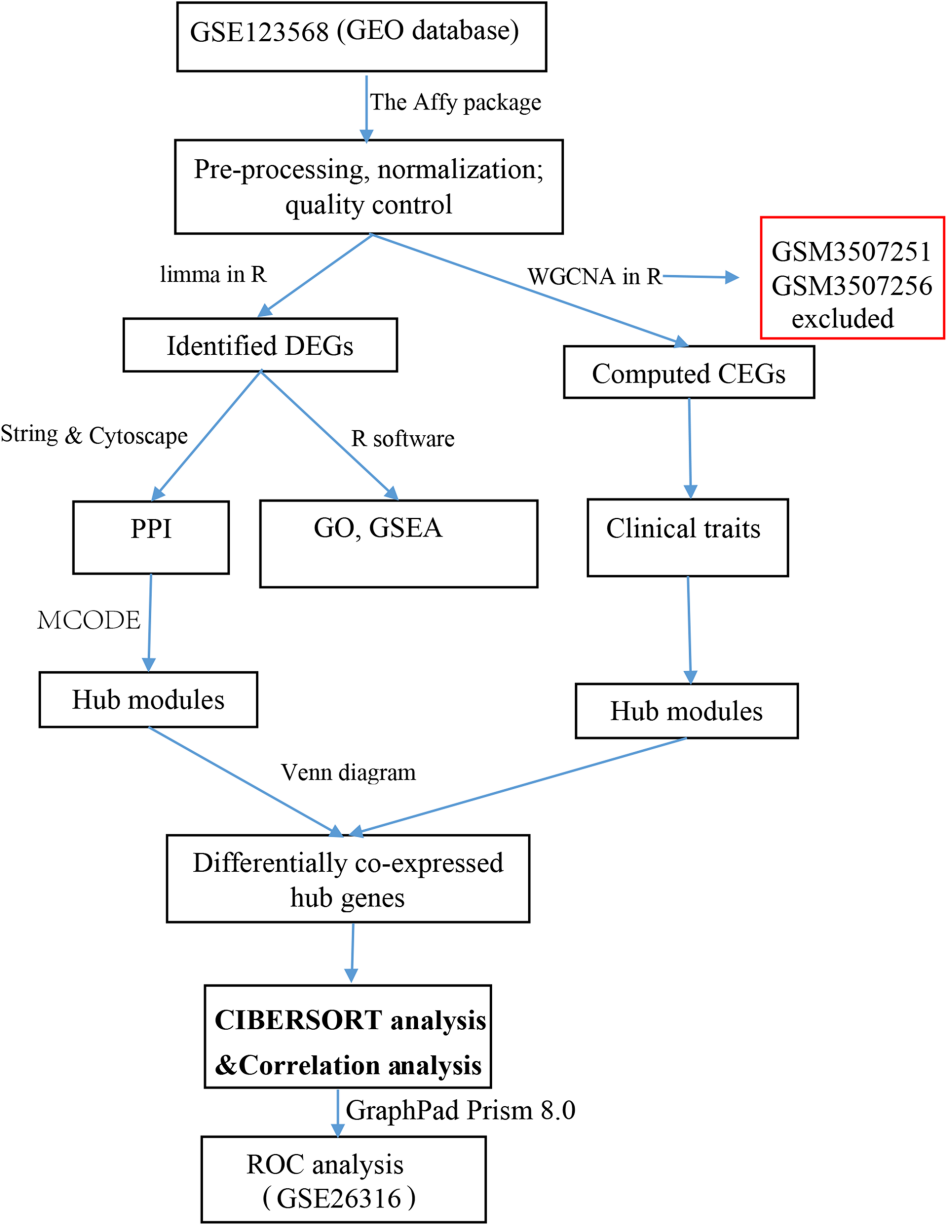
Flow diagram of the study. PPI: protein-protein interaction; DEGs: differentially expressed genes; CEGs: co-expressed genes; ROC: Receiver operating characteristic; GSEA: gene set enrichment analysis; WGCNA: weighted correlation network analysis

**Fig. 2:**
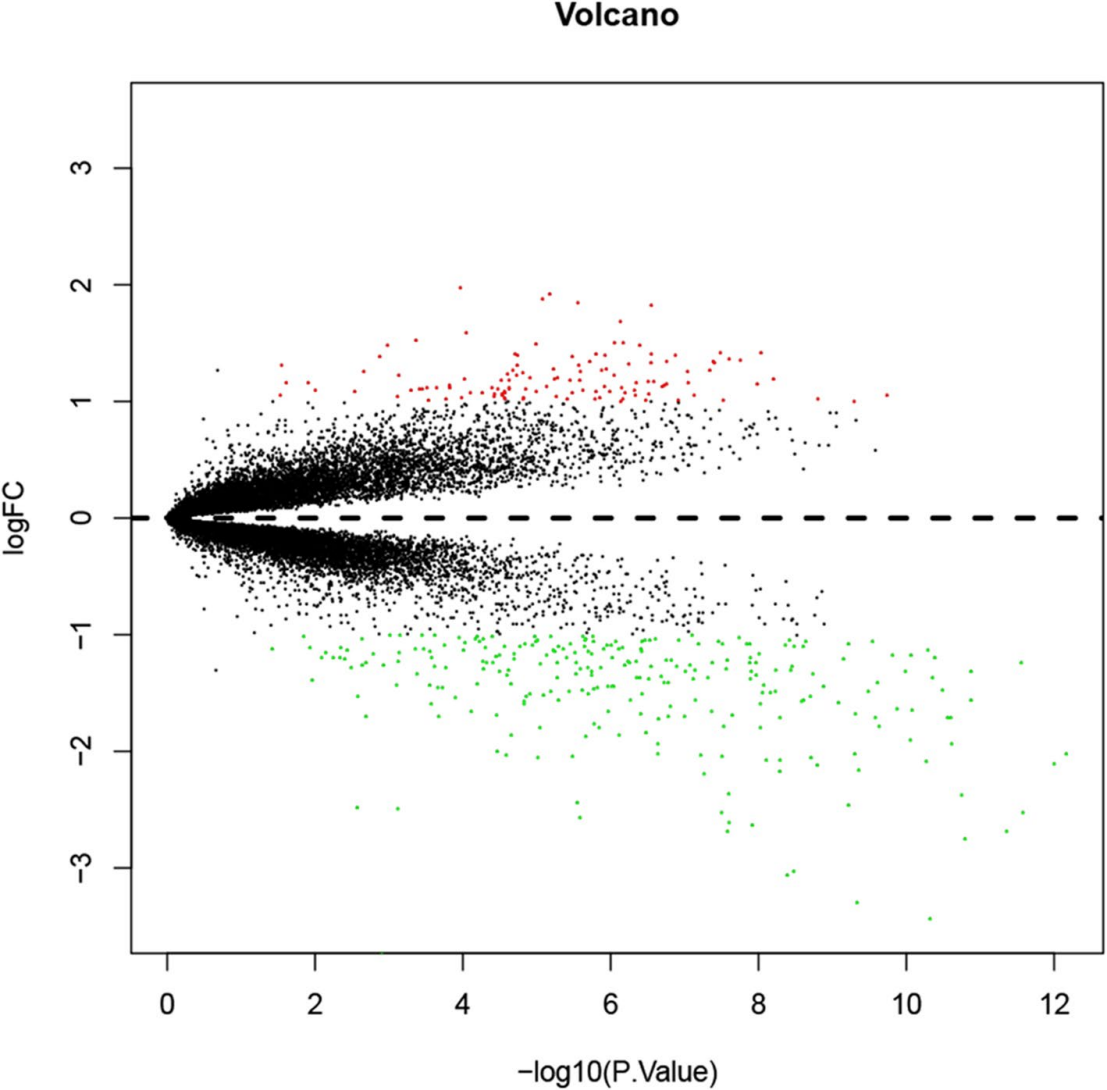
Volcano plot of the aberrantly expressed mRNAs in the GSE123568. Red: high expression; Green: low expression; Black dots: represent mRNAs that are not differentially expressed

Flow diagram of the study was provided in Fig. 1. DEGs were identified between the patients with SONFH and control samples after the microarray data GSE123568 preprocessed (Boxplots before and after data normalization were shown in Fig. S1). A total of 383 DEGs, including 119 upregulated genes and 264 downregulated genes, were detected (Table SI). The results were shown in the volcano map (Fig. 2, The clustering heatmap for the top 80 differential genes was shown in Fig. S2).

### The results of GO enrichment analysis and GSEA

**Fig. 3:**
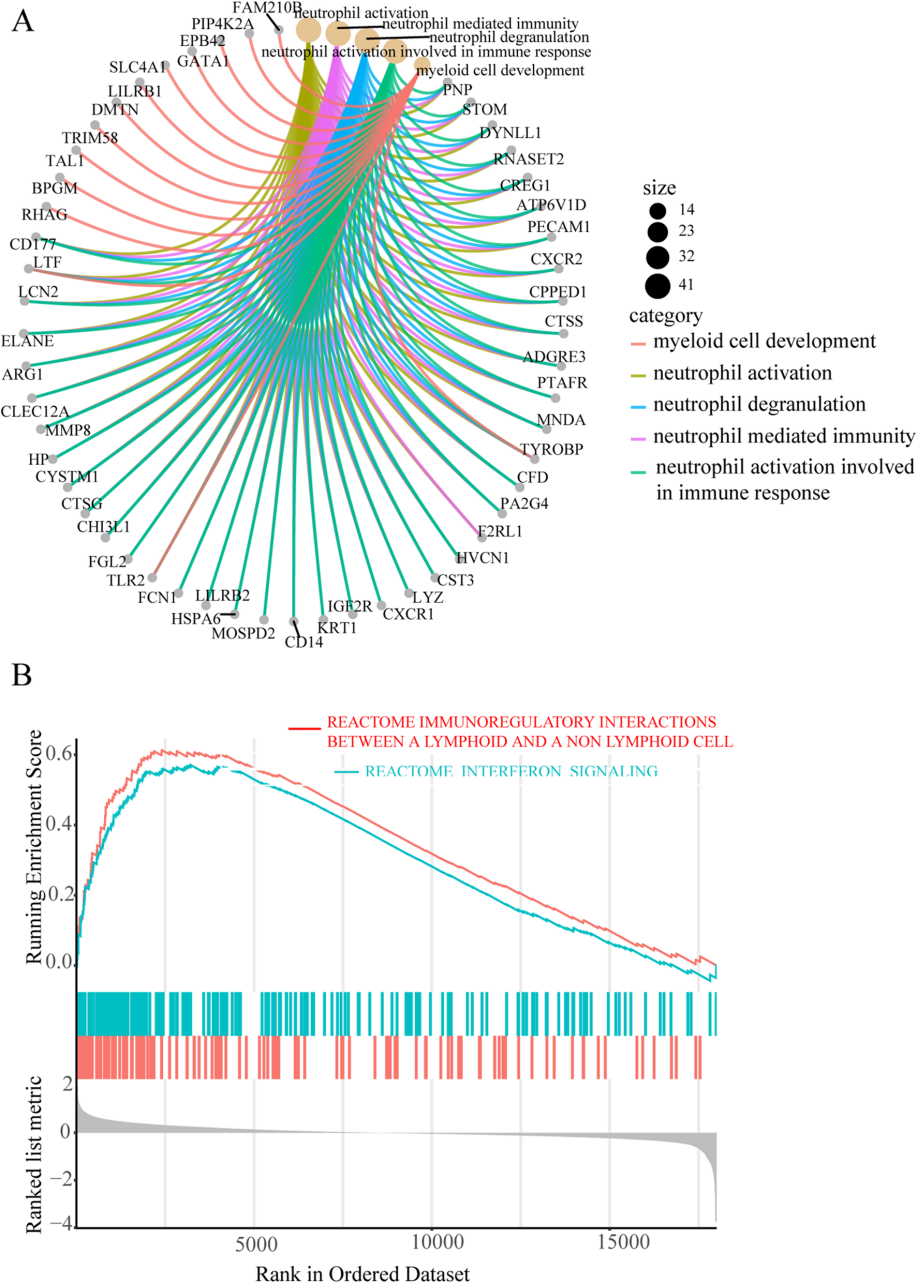
The results of GO enrichment analysis and GSEA. **A**: The results of the top five GO enrichment analysis and their related differentially expressed genes. **B**: Part of GSEA results

GO enrichment analysis results showed that DEGs were mainly related to neutrophil activation, neutrophil mediated immunity, neutrophil degranulation, and neutrophil activation involved in immune response, as shown in Fig. 3A. GSEA results revealed that the enriched reactome pathways mainly involved immunoregulatory interactions between a lymphoid and a non-lymphoid cell, neutrophil-degranulation, and interferon-signaling showed in Fig. 3B. Those results indicated that the immune response played an essential role in SONFH.

### Identification and characterization of SONFH-associated modules using WGCNA

**Fig. 4:**
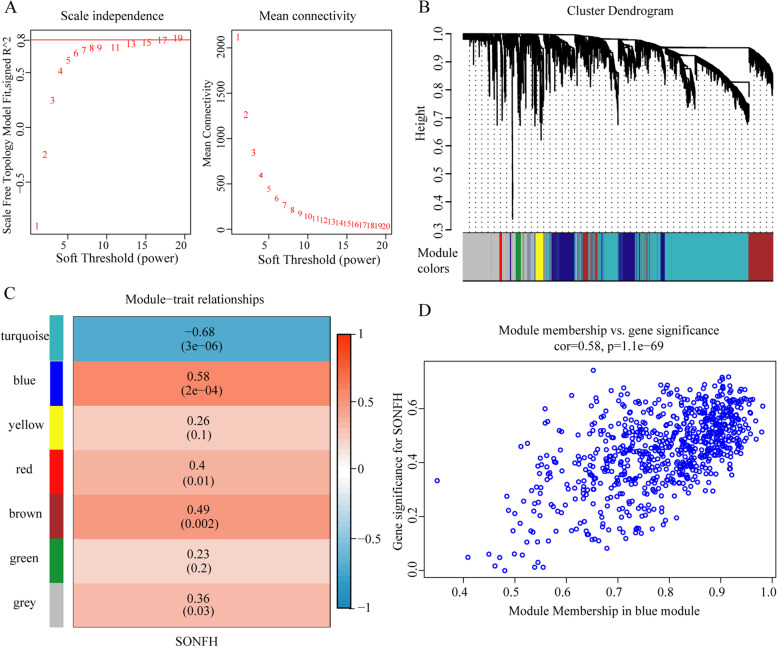
WGCNA processing for GSE123568. **A**: The power value of the adjacency matrix in WGCNA, β = 19, was selected as the best soft threshold for subsequent analysis. **B**: The cluster dendrogram with each color represented a co-expression module, and each branch represented one gene. **C**: Module-trait relationships, the numbers in the cell represented the correlation coefficient and corresponding *P*-value. **D**: Scatterplot of gene significance for SONFH vs. module membership in the blue module, one dot represented one gene. SONFH: steroid-induced osteonecrosis of the femoral head

The top 25% of genes (4699 genes) were obtained from GSE123568 with 40 samples based on variance analysis. Sample cluster analysis demonstrated that GSM3507251 and GSM3507256 samples were outlier samples, so they were deleted (Fig. S3). In our research, β =19 (scale-free *R*^2^ = 0.80) was set as the soft threshold to construct the co-expression network for a scale-free network (Fig. 4A. To satisfy a scale-free network topology, we choose the soft-threshold power β of eight with *R*^2^ = 0.80, as shown in Fig. S4). Then, we obtained seven gene co-expression modules using the dynamic tree cutting method (Fig. 4B). By relating those modules to clinical traits, we found that the blue module (*r* = 0.58, *p* = 2e-04), including 747 genes (Table S2), had the highest correlation with the trait of SONFH-stage (Fig. 4C). Finally, based on an intramodular analysis, genes in the blue module (cor = 0.58, *p* = 1.1e-69, Fig. 4D) for further investigation were characterized by high module membership and gene significance.

### Screening key diagnostic markers

**Fig. 5:**
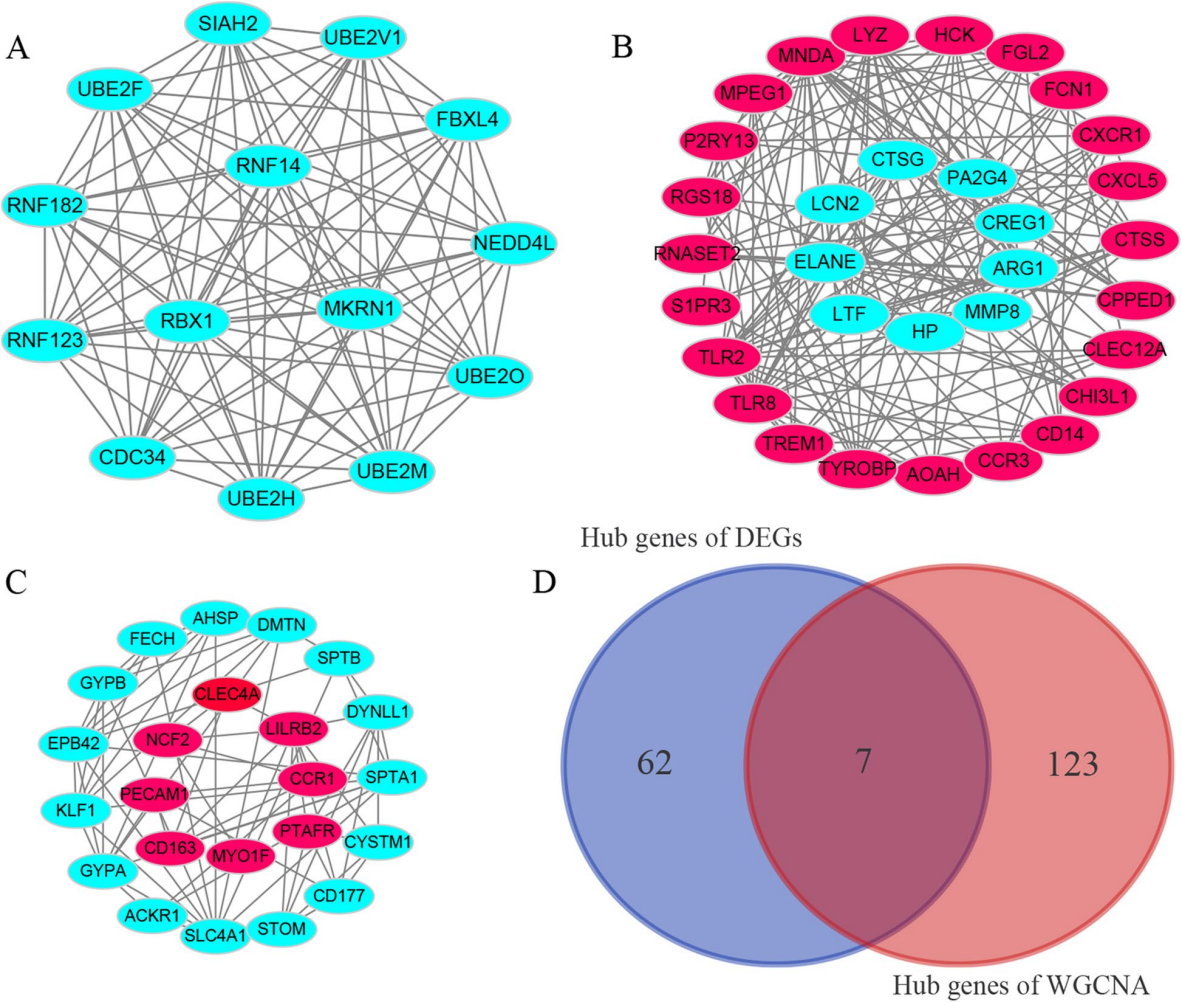
Screening key diagnostic markers. **A-C**: Based on the PPI network, the MCODE plug-in in Cytoscape was used to screen the key module with a score higher than 5. Pink represents up-regulated genes, while cyan represents down-regulated genes. **D**: Venn diagram shows the intersection of the key diagnostic markers obtained by the two algorithms. DEGs: differentially expressed genes; WGCNA: weighted correlation network analysis

To further identify the key diagnostic markers, we constructed the PPI network (as shown in Fig. S5) of DEGs using STRING and used the MCODE plug-in in Cytoscape to screen the key module with a score higher than 5. As a result, 69 genes in 3 modules were obtained, of which 31 were upregulated, and 38 were downregulated (Fig. 5A-C). Besides, after calculating GS and MM in the key blue module of WGCNA, 130 key genes were identified (a gene with MM > 0.9 and GS > 0.2 was defined as hub gene) (Tables S3, S4 and S5). Finally, seven key diagnostic markers TYROBP, TLR2, P2RY13, TLR8, HCK, MNDA, and NCF2 of SONFH obtained by the two algorithms, were overlapped (Fig. 5D).

### Immune infiltration analyses

**Fig. 6:**
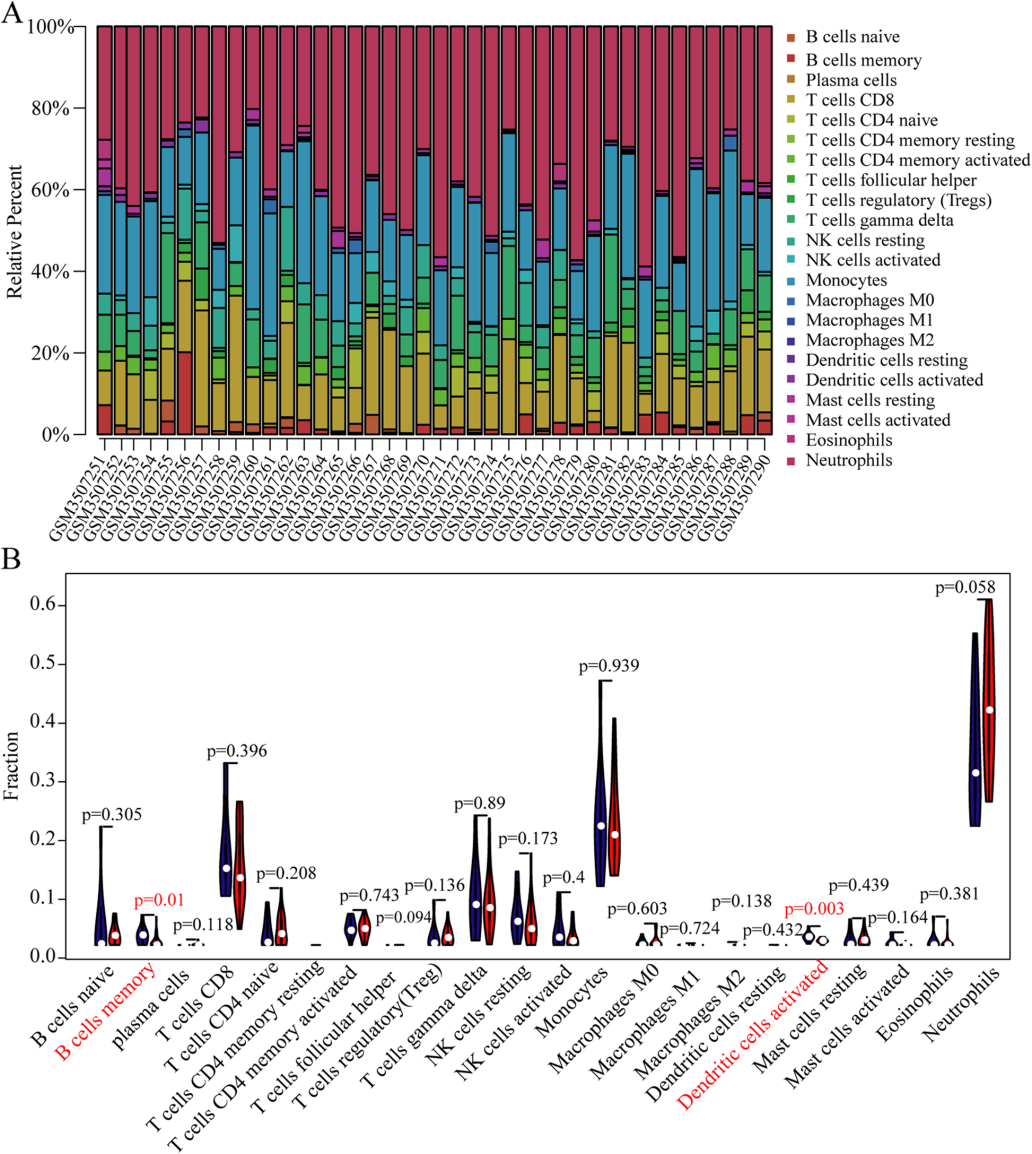
Analysis of immune cell infiltration. **A**: The relative percentage of 22 kinds of immune cells in GSE123568 datasets. **B**: The difference of immune infiltration between SONFH (the crimson color) and normal controls (the dark blue color). The red marks represent the difference in infiltration between the two groups of samples

According to the CIBERSORT algorithm, we first obtained the difference of 22 immune cells (All values of CD4 memory resting T cells were 0) in 10 healthy controls and 30 SONFH patients (Table S6, Fig. 6A). The correlation heatmap of those immune cells showed that macrophages M0 was positively correlated with plasma cells, macrophages M1 was positively correlated with monocytes, follicular helper T cells had a positive correlation with regulatory T cells and activated dendritic cells, and neutrophils had a negative correlation with T cells CD8 (Fig. S6, Table S7). Compared with healthy samples, memory B cells and activated dendritic cells in SONFH samples were relatively infiltrated less (Fig. 6B).

### Analysis of correlation between key diagnostic markers and immune infiltrating cells

**Fig. 7:**
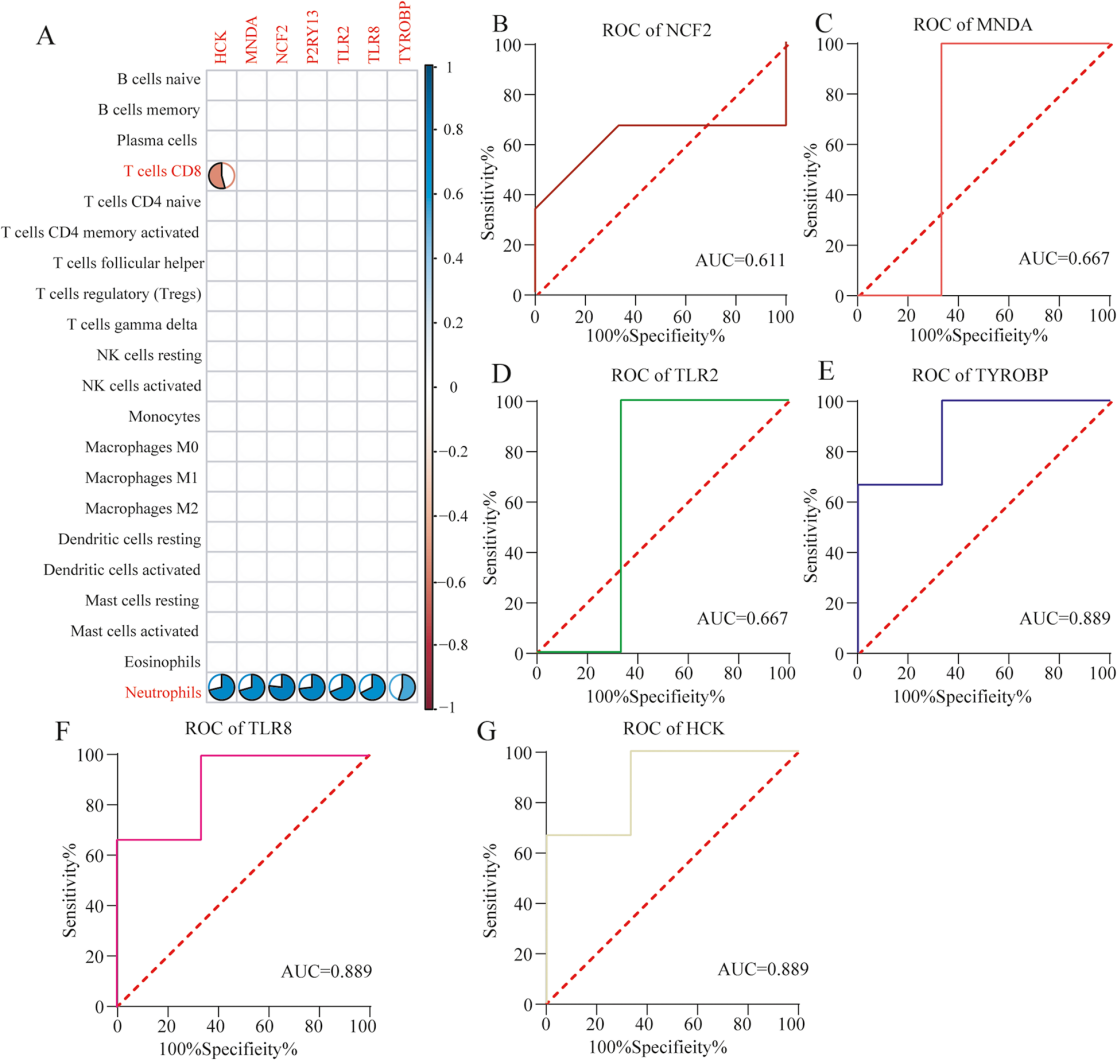
Verification of key diagnostic markers and their correlation with immune cells infiltration. **A**: Correlation between seven key diagnostic markers and infiltrating immune cells. The absolute value of the coefficient greater than 0.5 and *p* < 0.05 are displayed. The immune cells identified as related are represented in red, and the size of the colored area of the circle depicts the strength of the correlation. **B**-**H**: The ROC curve of the diagnostic efficacy verification. AUC: area under the curve

The results of Spearman correlation analysis (Table S8) revealed that HCK was negatively correlated with CD8 T cells (*r* = − 0.541, *p* = 0.043) and neutrophils were positively correlated with TYROBP (*r* = 0.549, *p* = 0.035), TLR2 (*r* = 0.689, *p* = 1.25e-04), P2RY13 (*r* = 0.731, *p* = 1.27e-05), TLR8 (*r* = 0.676, *p* = 2.38e-04), HCK (*r* = 0.712, *p* = 3.71e-05), MNDA (*r* = 0.706, *p* = 5.11e-05), and NCF2 (*r* = 0.768, *p* = 1.08e-06) (Fig. 7A).

### Verification of key diagnostic markers

To further test the diagnostic efficacy of seven key diagnostic markers, we validated it in the GSE26316 dataset. Except for P2RY13 (Interestingly, there was no expression data of the gene in this data matrix), the diagnostic efficiency of key diagnostic markers reached a high level (Fig. 7B-G), highlighting the ability of these key diagnostic markers for SONFH.

## Discussion

Although the pathogenesis of SONFH is not completely clear, recent studies have focused on apoptosis, lipid metabolism disorders, stem cell differentiation, osteoporosis, and so on [21]. Besides, the molecular mechanism of the interaction between bone and the immune system has been gradually revealed, such as osteoimmune molecule RANKL (receptor activator of NF-κB ligand) [22], which is also closely related to SONFH [23]. Therefore, more and more attention has been paid to the role of immune cells in the formation mechanism of SONFH. In this study, we identified seven SONFH diagnostic markers by bioinformatics analysis and tried to clarify further the relationship between these markers and immune cell infiltration, which was also helpful in exploring the role of immune cell infiltration in SONFH.

We identified a total of 383 DEGs from the serum expression profile dataset of SONFH. To explore DEGs’ function, GO enrichment analysis revealed that the DEGs were mainly involved in neutrophil activation, neutrophil mediated immunity, neutrophil degranulation, and neutrophil activation required in the immune response. Moreover, the results of GSEA showed that immunoregulatory interactions between a lymphoid and a non-lymphoid cell and neutrophil-degranulation pathways were significantly enriched. Jiang et al. [24] demonstrated a significant correlation between the percentage of neutrophils in peripheral blood and ONFH, which was consistent with our results and provided a new direction for further study of ONFH. WGCNA [18] can find the gene module associated with disease traits from the expression profile data and explore the crucial genes in the module. The MCODE plug-in [20] in Cytoscape can cluster and screen key gene modules in large PPI networks. In this study, we combined WGCNA and MCODE methods to identify TYROBP, TLR2, P2RY13, TLR8, HCK, MNDA, and NCF2 as diagnostic markers for SONFH. It should be emphasized that all diagnostic markers except P2RY13 were validated in the dataset GSE26316, which may be related to the fact that GSE26316 was obtained using femoral head samples rather than plasma samples. TYROBP is a protein involved in osteoclast differentiation, which is vital for bone absorption and has been proved to be a potential therapeutic gene for osteoporosis [25]. Besides, TYROBP is a critical regulator in the immune system, and the inactivation of TYROBP may lead to overproduction of pro-inflammatory and autoantibodies cytokines by macrophages [26]. Given the activation of osteoclasts in SONFH [27], we believe that TYROBP may regulate the pathological process of SONFH and related to immune cell infiltration. TLRs family plays a crucial role in the innate immune response, promoting inflammatory reactions through the NF-κB signal transduction and MAP kinase pathway [28]. It was reported that glucocorticoid induces inflammation related to the immune response through TLR pathways, which led to the occurrence of ONFH. For example, Okazaki et al. [29] demonstrated that the development of SONFH needs to be stimulated by TLR7 and TLR9, which can be promoted by inhibiting NF-Κb in rats. Studies show that there are high levels of crosstalk between TLR2 and TLR8, which can stimulate the secretion of inflammatory cytokines related to rheumatoid arthritis and other inflammatory diseases [30, 31]. In addition, Zhou et al. [32] revealed that activation of the TLR2 signaling pathway could promote angiogenesis and osteogenesis of bone marrow stromal cells. Therefore, we think that TLR2 and TLR8 may be involved in regulating the pathological process of SONFH.

P2RY13 is an extracellular ADP receptor that participates in purine energy transmission pathway, cholesterol metabolism, and regulation of bone homeostasis [33]. The previous study reported that bone marrow fat content in P2Y13 knockout mice increased, while bone formation decreased [34]. The main reason is that P2RY13 is the physiological determinant of bone marrow stromal cell differentiation and can control the balance of osteogenic and adipogenic differentiation [35], which is closely related to the mechanism of SONFH. HCK is a non-receptor tyrosine kinase, which plays an essential role in regulating cell growth, proliferation, phagocytosis of macrophages, differentiation, and pro-inflammatory secretion cytokines, apoptosis, and innate immune response [36]. For example, HCK promoted chondrocytes’ proliferation by activating Wnt and hedgehog signaling pathways in mice [37]. Another study demonstrated that HCK promoted bone homeostasis by controlling the recruitment of osteoclast precursors [38]. MNDA, which is mainly located in the nucleus, is a stress-induced protein that can promote the degradation of anti-apoptotic factor MCL-1 and apoptosis of myelocytes [39], and is the primary regulator of monocyte and granulocyte lineage [40]. Although little is known about the function of MNDA, it has been reported that MNDA can regulate bone marrow cell differentiation and neutrophil apoptosis [40, 41]. In addition, Wang et al. revealed that MNDA could be used as a diagnostic marker of periprosthetic joint infection [42]. NCF2 is an essential component of reactive oxygen species produced by phagocytes and is widely expressed in neutrophils, tumors, and other tissues [43]. Moreover, NCF2 plays an important role in innate immunity and diseases. Studies showed that NCF2 might be a potential marker of gastric cancer [43], and the high expression of NCF2 was associated with the risk of recurrence of renal cell carcinoma [44].

Then, we used CIBERSORT to evaluate SONFH immune infiltration and found that the decreased infiltration of memory B cells and activated dendritic cells may be related to the occurrence and development of SONFH. B lymphocytes are closely related to osteocytes because B lymphocytes need to be involved in osteoblast/stromal cells. Moreover, B lymphocyte progenitor cells may produce osteoclasts with normal function [45]. It was reported that autoantibodies produced by B cells provided diagnostic markers for autoimmune diseases such as lupus erythematosus and rheumatoid arthritis [46]. Additionally, another study showed that memory B cells play a role in regulating osteoclast formation in the pathogenesis of the periodontal disease [47]. Activated dendritic cells can migrate to lymphoid tissue to induce T cells to activate and differentiate into specialized cell subsets, and may also secrete various chemokine and interleukin to attract other immune cells to complete bone regeneration [48]. The increase of pro-inflammatory cytokines such as IL-17 can promote the differentiation of activated dendritic cells into functional osteoclasts in the presence of RANKL [49]. Finally, by analyzing the correlation between key diagnostic markers and immune cells, it was found that HCK was negatively correlated with CD8 T cells, and neutrophils were positively correlated with these key diagnostic markers, which was consistent with the previous study that neutrophils played an important role in SONFH [24]. It was important to note that although this study showed that varieties of immune cells may be associated with the occurrence and development of SONFH, these immune cells did not demonstrate a strong association with seven key diagnostic markers. Although some study showed such results [50], we speculate that it may be due to the difference in the algorithms and the fact that CIBERSORT analysis was based on limited genetic data. However, these key markers and their relationship with immune cell infiltration need further experimental investigation. Although there were few studies on the relationship between immune cell infiltration, such as memory B cells and activated dendritic cells and SONFH, it was reported that T and B lymphocytes play an important role in the pathogenesis of SONFH [5, 8]. Another study showed that the infiltration of M1 macrophages in the femoral head of SONFH was significantly increased and curcumin can inhibit the polarization of M1 macrophages from preventing osteocyte apoptosis and the development of SONFH [51], which provided an experience for us to explore the pathogenesis of SONFH and immune-related therapy for SONFH.

## Conclusions

In conclusion, we found that TYROBP, TLR2, P2RY13, TLR8, HCK, MNDA, and NCF2 may be key diagnostic markers of SONFH. But the functional verification of the experiment has not been carried out. Memory B cells and activated dendritic cells may be related to the occurrence and development of SONFH. Moreover, HCK was negatively correlated with CD8 T cells, and neutrophils were positively correlated with those key diagnostic markers. The immune-related mechanism of SONFH and the potential immunotherapy are worthy of further study.

## Supplementary Information

**Additional file 1.**

## Data Availability

The data used to support the findings of this study are included in the article, and all methods were performed in accordance with the relevant guidelines and regulations.
