# Supplementary material for: Identification of key biomarkers in steroid-induced osteonecrosis of the femoral head and their correlation with immune infiltration by bioinformatics analysis
Source: BMC Musculoskelet Disord. 2022 Jan 18;23:67. doi: 10.1186/s12891-022-04994-7 (PMC8767711; doi:10.1186/s12891-022-04994-7)
Supplement: Supplementary file 1 — Additional file 1. [file 12891_2022_4994_MOESM1_ESM.zip › legend of Figure S1~S6.docx]

**Figure S1.** Boxplots before and after data normalization.

**Figure S2.** The clustering heatmap for the top 80 differential genes. SONFH, steroid-induced osteonecrosis of the femoral head.

**Figure S3**. Sample cluster analysis.

**Figure S4**. Determination of soft-threshold power. When β is set at eight, the log-log plot of the network connectivity distribution produces a straight line.

**Figure S5**. The PPI network of DEGs.

**Figure S6.** The correlation heatmap of those immune cells.
