# Supplementary figures and images for: Identification of key biomarkers in steroid-induced osteonecrosis of the femoral head and their correlation with immune infiltration by bioinformatics analysis
Source: BMC Musculoskelet Disord. 2022 Jan 18;23:67. doi: 10.1186/s12891-022-04994-7 (PMC8767711; doi:10.1186/s12891-022-04994-7)

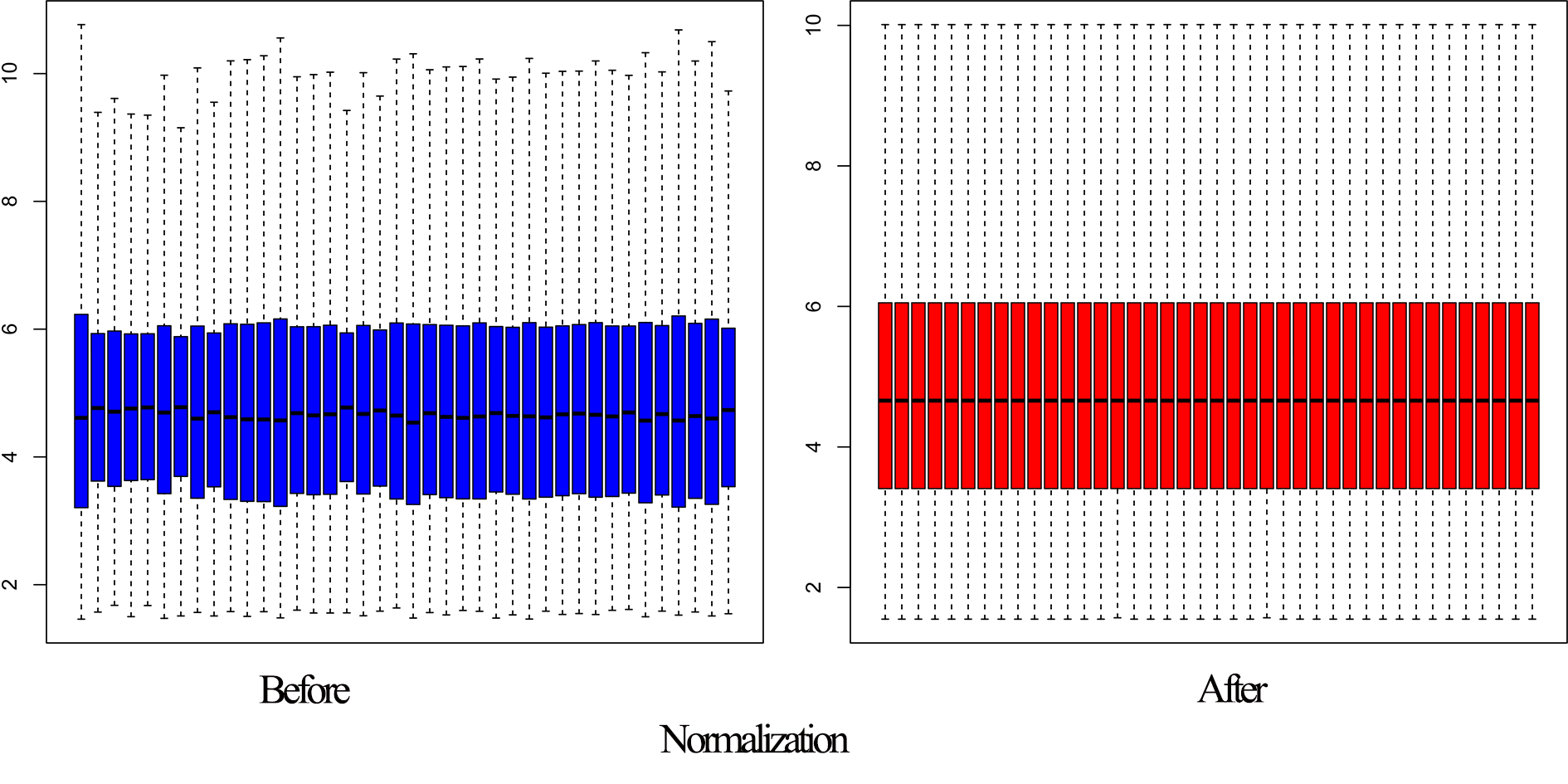

Supplement: Supplementary file 1 — Additional file 1. [file 12891_2022_4994_MOESM1_ESM.zip › Figure S1.tif]

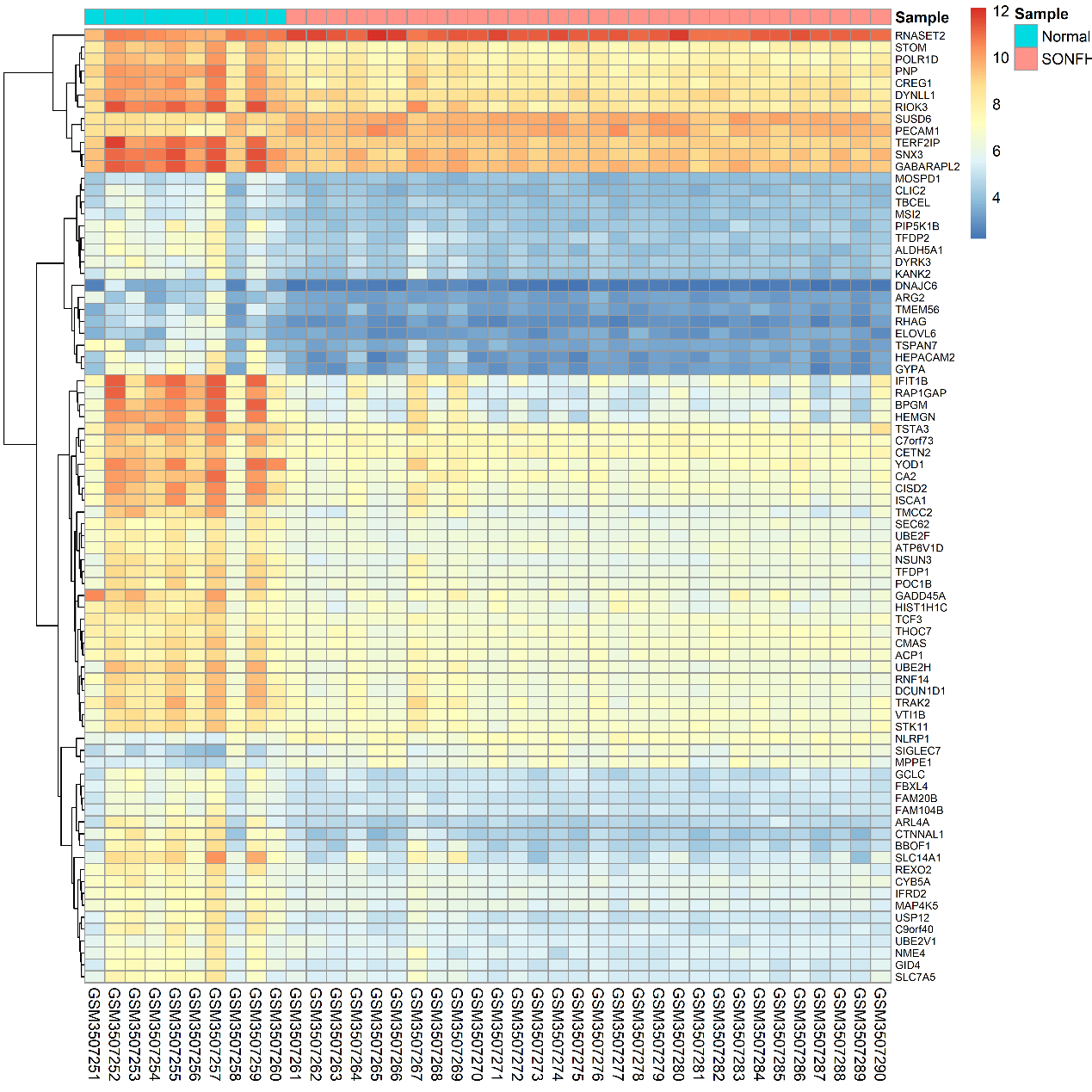

Supplement: Supplementary file 1 — Additional file 1. [file 12891_2022_4994_MOESM1_ESM.zip › Figure S2.tif]

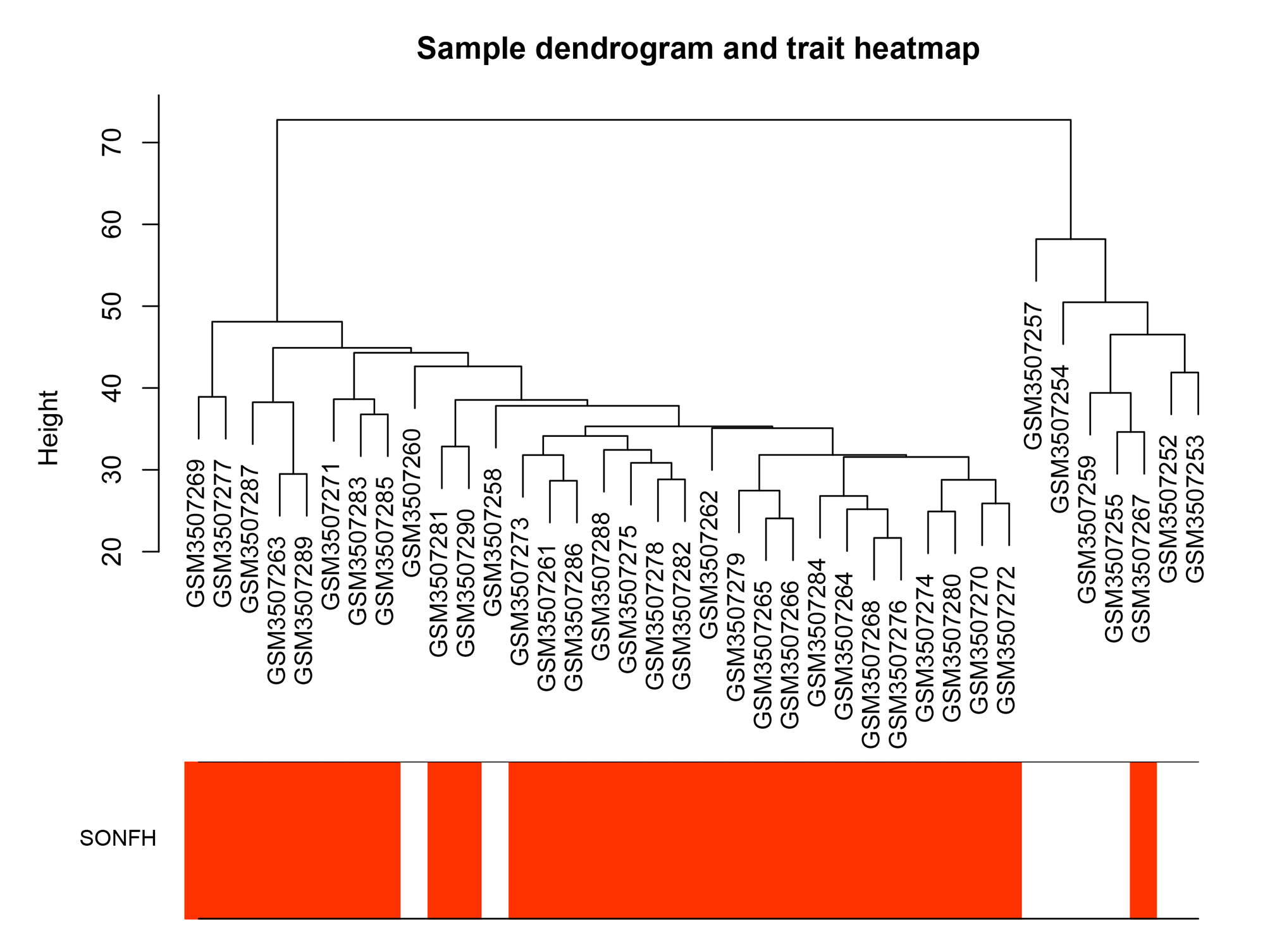

Supplement: Supplementary file 1 — Additional file 1. [file 12891_2022_4994_MOESM1_ESM.zip › Figure S3.tiff]

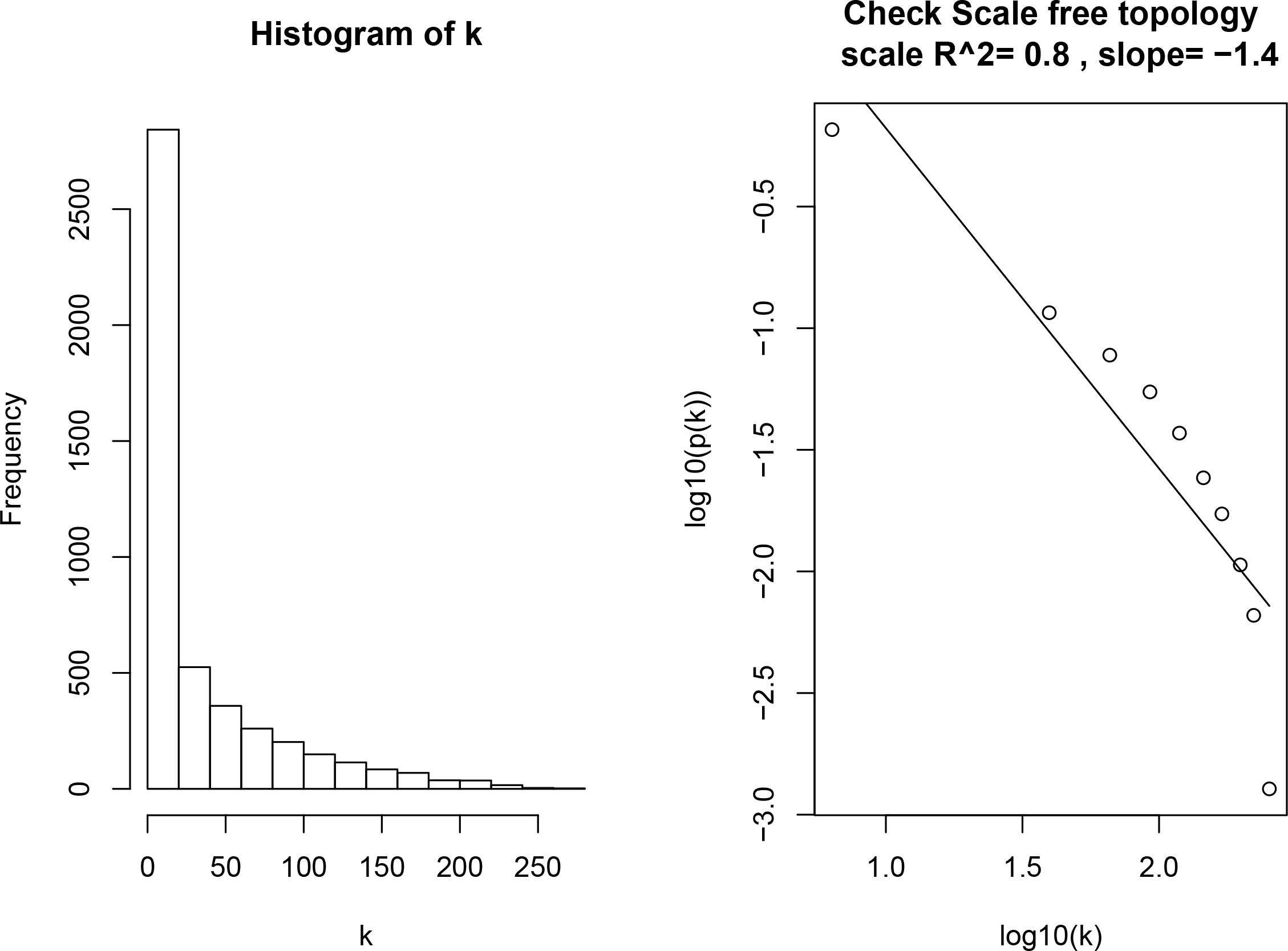

Supplement: Supplementary file 1 — Additional file 1. [file 12891_2022_4994_MOESM1_ESM.zip › Figure S4.tif]

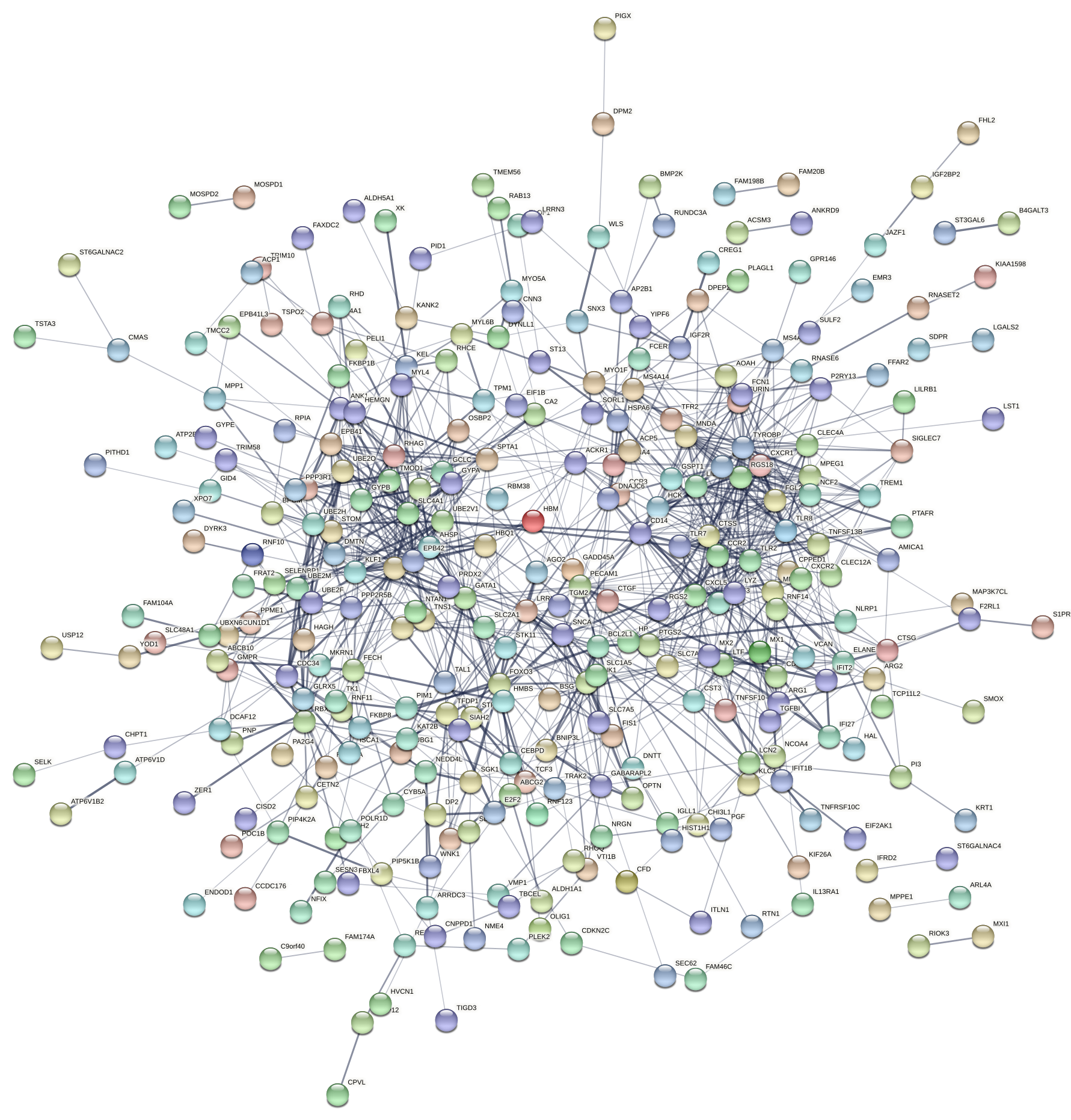

Supplement: Supplementary file 1 — Additional file 1. [file 12891_2022_4994_MOESM1_ESM.zip › Figure S5.png]

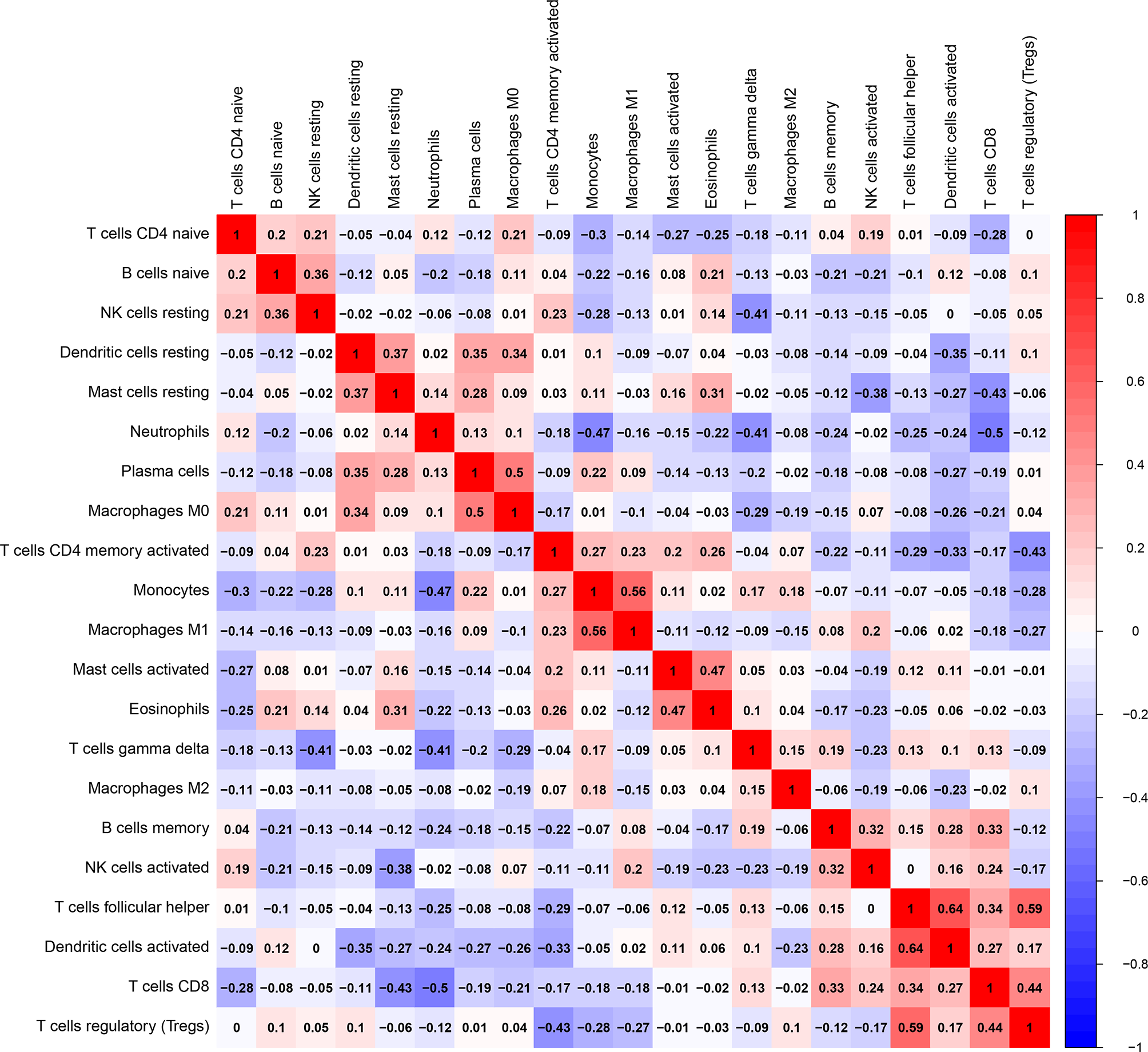

Supplement: Supplementary file 1 — Additional file 1. [file 12891_2022_4994_MOESM1_ESM.zip › Figure S6.tif]
